# A Highly Sensitive SPE Derivatization–UHPLC–MS Approach for Quantitative Profiling of Carotenoid-Derived Dialdehydes from Vegetables

**DOI:** 10.1021/acs.jafc.9b01749

**Published:** 2019-05-05

**Authors:** Jianing Mi, Kun-Peng Jia, Aparna Balakrishna, Qitong Feng, Salim Al-Babili

**Affiliations:** King Abdullah University of Science and Technology (KAUST), Biological and Environmental Sciences and Engineering Division, The BioActives Lab, Thuwal 23955-6900, Kingdom of Saudi Arabia

**Keywords:** food diapocarotenoids, diapocarotenoids chemical derivatization, diapocarotenoids SPE, diapocarotenoids profiling, UHPLC-MS

## Abstract

Oxidative cleavage of carotenoids leads to dialdehydes (diapocarotenoids, DIALs) in addition to the widely known apocarotenoids. DIALs are biologically active compounds that presumably impact human health and play different roles in plant development and carotenoid metabolism. However, detection of DIALs in plants is challenging due to their instability, low abundance, and poor ionization efficiency in mass spectrometry. Here, we developed a solid-phase extraction and derivatization protocol coupled with ultrahigh performance liquid chromatography−mass spectrometry for quantitative profiling of DIALs. Our method significantly enhances the sensitivity of DIAL detection with a detection limit of 0.05 pg/mg of dried food materials, allowing unambiguous profiling of 30 endogenous DIALs with C_5_ to C_24_ from vegetables. Our work provides a new and efficient approach for determining the content of DIALs from various complex matrices, paving the way for uncovering the functions of DIALs in human health and plant growth and development.

## INTRODUCTION

Carotenoids are isoprenoid pigments characterized by an extended conjugated double-bond system and synthesized by all photosynthetic organisms and many heterotrophic micro- organisms.^1,2^ Carotenoids exert vital functions in photo- synthetic organisms, protecting cells from photo-oxidation and contributing to the light-harvesting process. In addition, they are important nutrients, conferring their bright colors to many fruits and flowers.^3−5^ Several carotenoids present in fruits and vegetables act as provitamin A, providing around 82% of dietary vitamin A in developing countries.^6,7^ In plants, carotenoids are a precursor of hormones, i.e., abscisic acid and strigolactone, and several regulatory metabolites, such as β- cyclocitral, mycorradicin, and the recently discovered anchor- ene and zaxinone.^8−14^ In fungi, carotenoids are metabolized into retinoids and the pheromone trisporic acid.^15^ All of these carotenoid derivatives are formed by oxidative cleavage of double bonds in the carotenoid backbone, which yields carbonyl products known as apocarotenoids and diapocar- otenoids (dialdehydes, DIALs). This widespread metabolic process is generally catalyzed by carotenoid cleavage dioxygenases (CCDs) or triggered nonenzymatically by reactive oxygen species.^16,17^

DIALs have attracted attention mainly as precursors of bixin and crocin, two pigments accumulated in annatto fruits and saffron flowers, respectively.^18−20^ However, several studies indicate that DIALs have regulatory functions in plants and humans. For instance, it was very recently reported that anchorene, a C_10_ DIAL, is a signaling molecule that regulates root development in *Arabidopsis* and rice.^13^ In addition, studies on rosafluene, a C_14_ DIAL, and its structural isomer demonstrated that they inhibit the activity of nuclear factor kappa B in bone and cancer cells and reduce the growth of breast and prostate cancer cells.^21,22^ These results indicate the importance of DIALs for nutrition and plant science.

Several DIALs have been identified as in vitro products of plant and cyanobacterial CCDs. For instance, *all*-*trans*-4- methylocta-2,4,6-trienedial (C_9_) and *all*-*trans*-2,6-dimethyloc- ta-2,4,6-trienedial (C_10_) were identified as products of the rice zaxinone synthase and the cyanobacterial apocarotenoid cleavage oxygenases (SynACO and NosACO), respec- tively.^14,23,24^ However, the most striking enzymes in this regard are the tomato CCD1A and CCD1B, which show relaxed apocarotenoid substrate specificity in vitro, converting a wide range of apolycopenals derived from the tomato pigment lycopene into a series of DIALs with different carbon chains.^25^

Due to their instability and presence at low concentrations, the detection of DIALs has been mainly restricted to high- abundant DIALs produced enzymatically in in vitro studies. For example, *all*-*trans*-4-methylocta-2,4,6-trienedial produced from an in vitro assay was identified by using LC−MS after derivatization with *O*-(2,3,4,5,6-pentafluorobenzyl) hydroxyl- amine hydrochloride,^14^ where the long derivatization process time (1 h at 35 °C) increases the risk of DIALs degradation. The in vitro assay product *all*-*trans*-2,6-dimethylocta-2,4,6- trienedial was identified by using GC−MS.^24^ However, understanding the impact of DIALs consumed in food on human health and elucidating their function in plant development and carotenoid metabolism require an analytical method that enables sensitive and reliable determination of these compounds, which is up to now not available. Here, a SPE-chemical derivatization−UHPLC−MS method was de- veloped for analyzing carotenoid-derived DIALs from vegeta- bles, which paves the way for investigating the biological activity of this class of DIALs.

## EXPERIMENTAL SECTION

### Materials and Reagents

**Figure 1:**
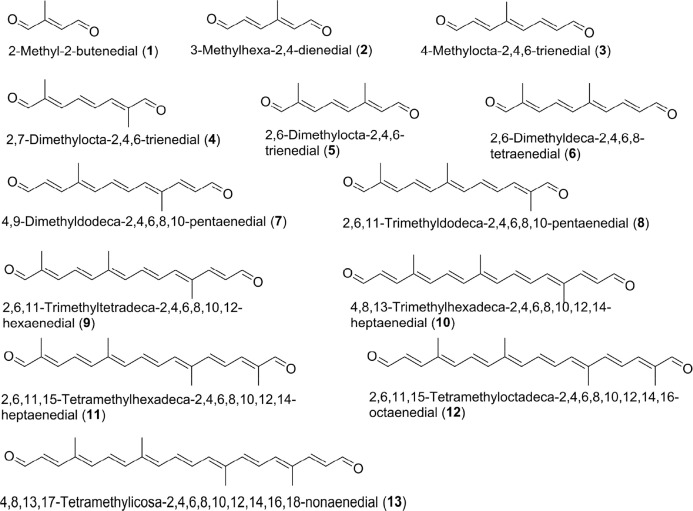
Structures of representative carotenoid-derived DIALs.

LC−MS grade methanol (MeOH), acetonitrile (ACN), 2-propanol (IPA), formic acid (FA), and butylated hydroxytoluene (BHT, 99%) were purchased from Sigma- Aldrich (Taufkirchen, Germany). LC−MS grade water; HPLC-grade *n*-hexane, ethyl acetate, and acetone were purchased from VWR International, LLC. (Pennsylvania, US). DIAL standards including *all*- *trans*-2,7-dimethylocta-2,4,6-trienedial (4, Figure 1), *all*-*trans*-2,6-dimethylocta-2,4,6-trienedial (5), *all*-*trans*-2,6,11-trimethyldodeca- 2,4,6,8,10-pentaenedial (8), and *all*-*trans*-2,6,11,15-tetramethylhexa- deca-2,4,6,8,10,12,14-heptaenedial (11) and apocarotenoid standards including *all*-*trans*-3-OH-β-apo-11-carotenal (OH-Apo11), *all*-*trans*- 3-OH-β-apo-15-carotenal (OH-Apo15), and *all*-*trans*-3-OH-β-apo-12′-carotenal (OH-Apo12′) were obtained from Buchem B.V. (Apeldoorn, Netherlands). *N^2^,N^2^,N^4^,N^4^*-Tetraethyl-6-hydrazinyl- 1,3,5-triazine-2,4-diamine (T3) was bought from Chemspace. Silica gel SPE columns (500 mg/3 mL) were purchased from Thermo Scientific. *D*_6_-*all*-*trans*-2,7-Dimethylocta-2,4,6-trienedial (*D*_6_-4) was synthesized by Dr. Magnus Rueping according to the protocol.^13^ Several vegetables including tomato, carrot, yellow pepper, spinach, and sweet potato were obtained from the supermarket.

### Sample Preparation

A DIAL standards mixture solution I (including 4, 5, 8, and 11; 0.1 μg/mL each standard) was prepared in acetone for optimizing the derivatization and SPE procedures. DIAL standards mixture solution II (including 4 and 5; 1 μg/mL each standard) was prepared in acetone for quantitative method validation. *D*_6_-4 stock solution (Internal standard, 1 μg/mL) was prepared in acetone for quantitative method validation and quantitation of DIALs.

Vegetables were lyophilized, powdered, and stored at −20 °C. BHT (0.1%) was used to minimize oxidative degradation of carotenoid-derived DIALs during the whole procedure.^26^ Approx- imately 10 mg of vegetables powder (tomato *n* = 3, carrot *n* = 4, yellow pepper *n* = 4, spinach *n* = 4, and sweet potato *n* = 4) spiked with 0.5 ng of *D*_6_-4 was extracted with 1 mL of ACN containing 0.1% BHT in an ultrasound bath (Branson 3510 ultrasonic bath) for 15 min, followed by centrifugation for 8 min at 4000 rpm at 4 °C. The supernatant was collected, and the pellet was re-extracted with 1 mL of the same solvent. Then the two supernatants were combined and dried by N_2_. The residue was redissolved in 500 μL of *n*-hexane as the loading sample for the following SPE purification.

### Silica Gel SPE Enrichment

In order to enrich DIALs and to reduce the matrix interference, different combinations of *n*-hexane and ethyl acetate including 90:10, 80:20, 70:30, and 60:40 (v/v) were tested as elution solvent. Best results were obtained with *n*-hexane/ ethyl acetate, 70:30, v/v, which was then used to elute DIALs. The loading sample was run through a silica gel SPE column (500 mg/3 mL) preconditioned with 3 mL of ethyl acetate and 6 mL of *n*-hexane. After being washed with 3 mL of *n*-hexane, DIAL-enriched fraction was eluted in 6 mL of *n*-hexane/ethyl acetate (70:30, v:v) (Fraction 1, F1) and dried by N_2_. Hydroxylated apocarotenoids were eluted in 3 mL of MeOH (F2). The resulting DIAL-enriched fraction was redissolved in 200 μL of ACN with 0.025% BHT and transferred to 2 mL tube, followed by the dryness with N_2_.

### DIALs Derivatization

DIALs were derivatized according to the protocol described previously with minor modification.^27^ A 50 μL sample of T3 solution (2 mg/mL) in MeOH with 1% FA was added to the extract, followed by an incubation at 37 °C for 15 min under shaking. The sample solution was then diluted to 100 μL with 1% formic acid in MeOH and filtered through a 0.22 μm filter before LC−MS analysis.

### UHPLC-Q-Orbitrtap MS Detection

Analysis of derivatized DIALs was performed on a Dionex Ultimate 3000 UHPLC system coupled with a Q-Orbitrap-MS (Q-Exactive plus MS, Thermo Scientific) with a heated-electrospray ionization source. Chromato- graphic separation was carried out on an ACQUITY UPLC BEH C_18_ column (100 × 2.1 mm, 1.7 μm, Waters) with an UPLC BEH C_18_ guard column (5 × 2.1 mm, 1.7 μm, Waters) maintained at 35 °C. The optimized mobile phases A (H_2_O/ACN/FA, 90:10:0.2, v/v/v) and B (ACN/IPA/FA, 90:10:0.2, v/v/v) were employed for eluting DIALs with the optimized gradient program: 0−15 min, 20% B to 100% B; 15−20 min, 100% B; 20−21 min, 100% B to 20% B; 21−25 min, 20% B. The flow rate was 0.2 mL/min, and the injection volume was 10 μL. The optimized MS parameters were as follows: sheath gas flow rate of 30 arbitrary units, auxiliary gas flow rate of 5 arbitrary units, spray voltage of 4.0 kV, capillary temperature of 350 °C, auxiliary gas heater temperature of 400 °C, S-lens RF level of 50, and resolution of 70,000. For MS/MS measurements, NCE of 20 eV was used.

### Method Validation

The calibration linearity for DIALs was evaluated by spiking *D*_6_-4 at the following amounts, 0.001, 0.005, 0.01, 0.05, 0.1, 0.5, 1, 5, and 10 ng, to samples prior to extraction (*n* = 3). Regression analysis was used to calculate linear regression equations for *D*_6_-4 using GraphPad Prism 5 software. Limit of detection (LOD) and limit of quantitation (LOQ) were defined as the lowest concentration when signal-to-noise ratios of about 3 and 10 were obtained, respectively. The method mean recovery was examined by comparing signal response of *D*_6_-4 (0.5 ng) spiked to samples before and after the extraction and SPE purification (*n* = 6). Intra- and interday precisions of the quantitative procedure were determined on the basis of the results of five analyses of samples within a day and nine analyses of samples on three consecutive days. All precision was obtained by calculating the relative standard deviations (RSDs) for the levels of endogenous DIALs in samples. Different amounts of endogenous 4 and 5 (0.05−10 ng), spanning from <10% to >10 folds of endogenous 4 and 5, were spiked into the samples with internal standard, and the determined amount was calculated as the difference of determined amount of sample with and without spiking 4 and 5. The correlation between the added and determined amount of 4 and 5 was examined for accuracy.

### Data Processing and Statistical Analysis

This approach was applied to vegetables including tomato, carrot, yellow pepper, spinach, and sweet potato. The quantitation of DIALs was calculated as follows: amount _[target DIAL]_ = area_[target DIAL]_/area_[spiked D6‑4]_ × amount_[spiked D6‑4]_/dry weight. Statistical analyses of the amounts of different DIALs were performed using the GraphPad Prism 5 software. Student’s t test was used to analyze data and compare groups.

## RESULTS AND DISCUSSION

### Improvement of Detection Sensitivity of DIALs by T3-Derivatization

**Figure 2:**
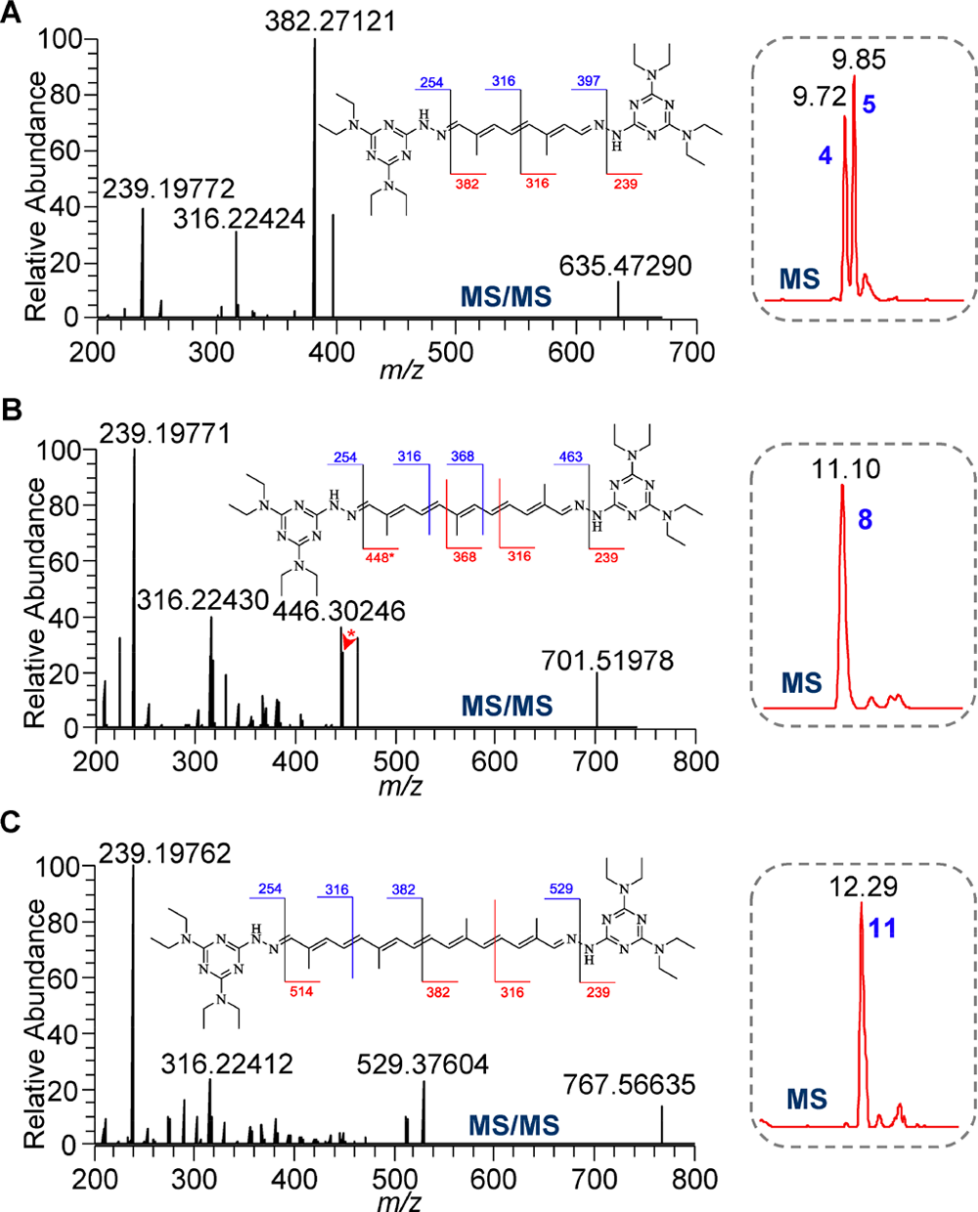
MS/MS spectra of T3-derivatized 5 (A), 8 (B), and 11 (C) standards. Insets: extracted ion chromatograms (EICs) and proposed cleavages of T3-derivatized DIALs.

On the basis of results obtained by Tie et al.^27^ showing efficient derivatization of fatty aldehydes by T3 under mild reaction conditions, a T3-derivatization method was developed for analyzing DIALs (Figure S1A). First, the reaction conditions, including reaction solvent, reaction temperature, and reaction time, were validated using the representative DIAL standards 5, 8, and 11 (Figure S1B). The results showed that this mild reaction was complete within 15 min at 37 °C. Comparison of the ionization efficiency of DIALs with and without derivatization (Figure S1C) demonstrated that T3-derivatization remarkably improved detection sensitivity of DIALs with short (C_10_) and middle carbon-chain (C_15_). It is worth noting that the short carbon- chain DIALs (4 and 5) could not be detected without derivatization. For long carbon-chain (C_20_) DIALs, no significant increase in sensitivity after T3-derivatization. Next, four representative DIAL standards were selected to optimize UHPLC and MS conditions. The results obtained showed that adding 0.2% FA to the mobile phases is crucial for improving chromatographic separation of DIAL isomers (i.e., 4 and 5) (Figure 2A). Furthermore, the ionization efficiency of T3- derivatized DIALs was increased remarkably by optimizing HESI source conditions of MS, for instance by increasing capillary temperature and Aux gas heater temperature, or by decreasing sheath gas and Aux gas flow rate (Figure S2).

The characterization of T3-derivatized DIAL standards allowed the identification of endogenous DIALs from vegetables, based on high resolution MS/MS data (Figure 2). The accurate mass of targeted compounds was obtained from high-resolution MS, providing its confidence chemical formula. The feature ions from the targeted MS/MS spectrum represent the basic structure backbone of the compound candidate. For example, The MS/MS pattern of T3-derivatized DIALs (Figure 2) showed that the ion at *m*/*z* 239 ([T3 − NH_2_ + H]^+^) resulted from the cleavage of nitrogen−nitrogen single bond; and that the ions at *m*/*z* 397 and *m*/*z* 382 (Figure 2A), *m*/*z* 463 and *m*/*z* 448 (Figure 2B), and *m*/*z* 529 and *m*/*z* 514 (Figure 2C) reflected the fragmentations of mono-T3- derivatized 5, 8, and 11 by the loss of one T3 moiety, respectively. A cleavage of the carbon−carbon bond in conjugated carbon chain gave rise to the *m*/*z* 316 ion, which reflects a T3-Valylene moiety of T3-derivatized DIALs.

### Minimizing Endogenous Matrix Metabolites by SPE

**Figure 3:**
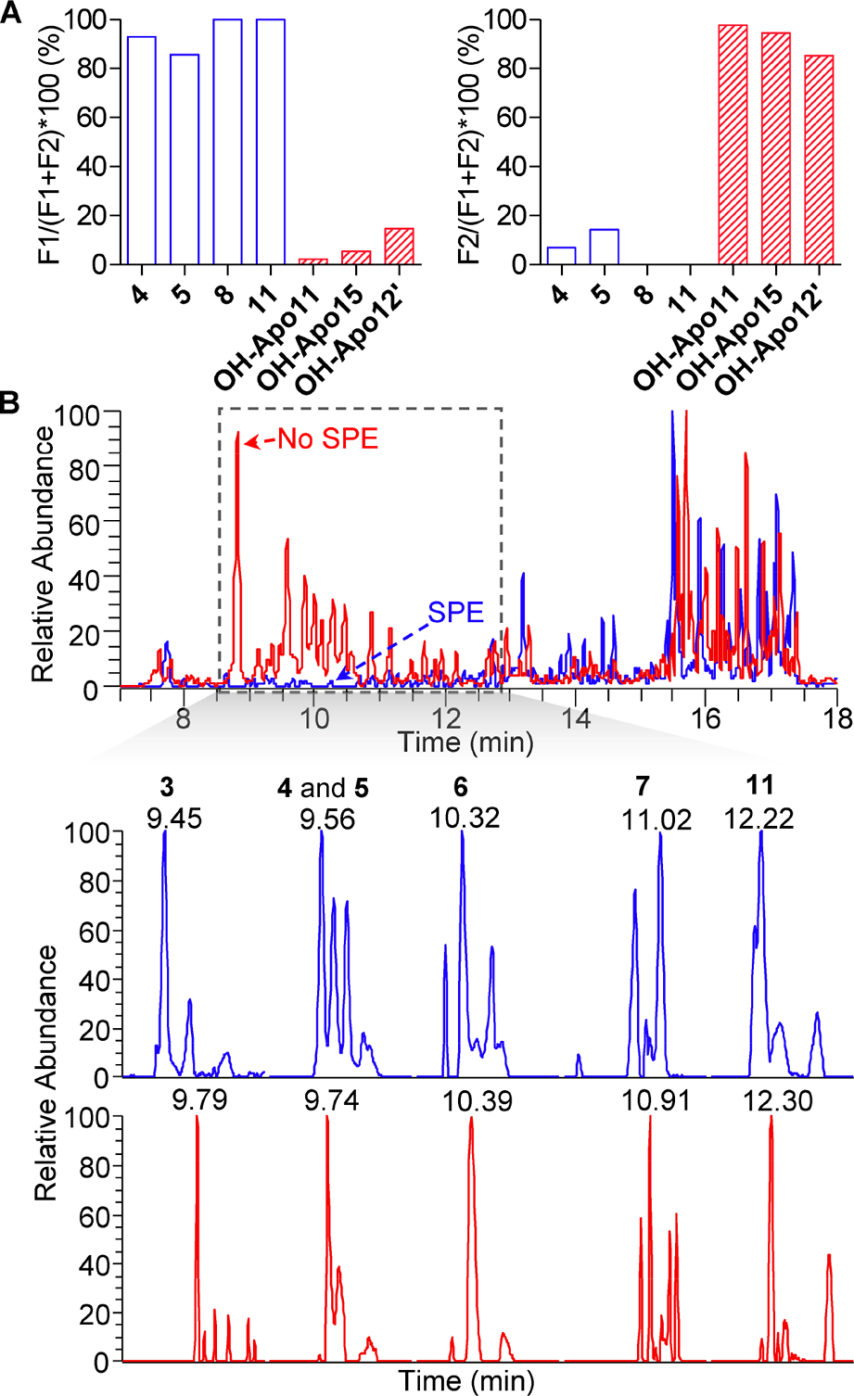
Effect of the SPE purification on the detection of T3- derivatized DIALs. (A) Optimal SPE purification procedure. (B) Total ions chromatograms (TICs) of tomato samples with (blue) and without (red) SPE purification (top) and EICs of representative endogenous T3-derivatized DIALs with (blue) and without (red) SPE purification (bottom).

Similar to other endogenous metabolites, a complicated matrix makes the characterization of DIALs difficult. Due to the T3 selectivity, treatment with this reagent leads to derivatization of all metabolites with carbonyl groups, including high-abundant hydroxylated apocarotenals (i.e., OH-Apo11, OH-Apo15, OH- Apo14′, and OH-Apo12′) (Figure S3). Analysis of T3- derivatized DIALs was further impeded by overlaps with T3- derivatized apocarotenoids in chromatographic retention time. To minimize the content of endogenous metabolites with carbonyl groups other than DIALs and reduce background signals, a SPE procedure was developed using representative DIAL and apocarotenoid standards, including 4, 5, 8, 11, OH- Apo11, OH-Apo15, and OH-Apo12′. Results showed that more than 80% of DIALs covering C_10_ to C_20_ carbon-chainswere successfully enriched in *n*-hexane/ethyl acetate (70:30, v/ v) fraction (F1), while only less than 20% apocarotenoids with carbon-chains ranging from C_15_ to C_25_ coeluted with DIALs (Figure 3A). Next, this optimized SPE method was applied in LC−MS analysis of DIALs in tomato fruits. As shown in Figure 3B, there is a significant increase in detection sensitivity and accuracy of these compounds with a low ion suppression effect due to an obvious reduction in background signals in the retention time range of T3-derivatized DIALs (8.5−13.5 min) in DIAL-enriched fraction (Figure 3B).

### Endogenous DIAL Identification in Vegetables

**Figure 4:**
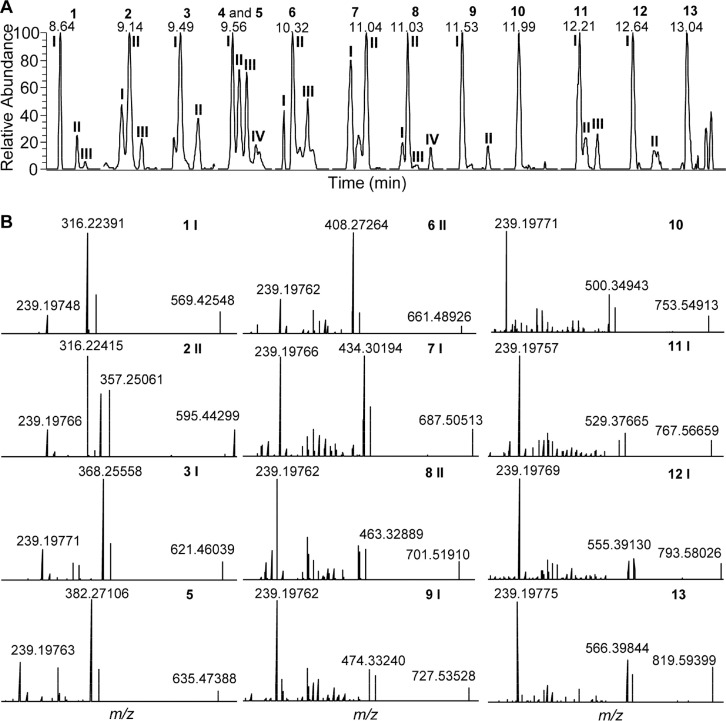
Identification of DIALs from tomato fruits by using UHPLC−MS: (A) EICs of 30 endogenous T3-derivatized DIALs from tomato; (B) high-resolution MS/MS spectra of representative endogenous T3-derivatized DIALs with NCE of 20 eV.

Next, the global profiling of DIALs in vegetables was performed using our new approach. For this purpose, the corresponding DIAL-enriched extracts were derivatized using T3 and analyzed by using UHPLC−MS. Thirty potential DIALs were identified according to their accurate masses and high- resolution MS/MS data showing patterns similar to those of DIAL standards (Figures 2 and 4, Table 1). Stratagems for the assignment of endogenous DIALs from vegetables are summarized: (1) DIAL candidates were selected by personal database matching on the basis of the accurate mass obtained from high-resolution MS (Table 1). (2) The MS/MS data were used to validate the assignment of DIAL candidates; for example, the feature ion at *m*/*z* 239 should occur in the MS/ MS spectra of all DIALs candidates, and clear fragmentations (i.e., *m*/*z* 368 for 3 I, *m*/*z* 408 for 6 II, *m*/*z* 474 for 9 I, and *m*/ *z* 555 for 12 I) exist in MS/MS spectra of various of DIALs, reflecting moieties with different carbon-chain via losing a T3 (Figure 4B). (3) Identification of DIALs was confirmed by using representative available DIAL standards (Figure S4A). (4) The UHPLC chromatographic retention time rule was employed to predict the assignment of DIALs candidates without standards,^28^ for example, the retention times of T3- derivatized DIALs with different carbon chains exhibited a clear relationship with the length of the corresponding carbon chain (*r*^2^ = 0.9989 and *r*^2^ = 0.9954) (Figure S4B).

**Table 1:** Identification of 30 DIALs from Foods by Using UHPLC−MS/MS

DIALs	*t*_R_ (min)	formula (DIALs)	Formula (DIALs +T3)	exptl [M +H]^+^ (*m*/*z*)	theor [M + H]^+^ (*m*/*z*)	error (ppm)	MS/MS fragmentations	notes
**1 I*^a^***	8.64	C_5_H_6_O_2_	C_27_H_48_N_14_	569.42554	569.42591	−0.66	331.23, 316.22, 254.21, 239.20	tentatively identified*^b^*
**1 II**	8.98	C_5_H_6_O_2_	C_27_H_48_N_14_	569.42542	569.42591	−0.88	331.23, 316.22, 254.21, 239.20	tentatively identified
**1 III**	9.14	C_5_H_6_O_2_	C_27_H_48_N_14_	569.42542	569.42591	−0.88	331.23, 316.22, 254.21, 239.20	tentatively identified
**2 I**	9.03	C_7_H_8_O_2_	C_29_H_50_N_14_	595.44153	595.44156	−0.06	357.25, 342.24, 316.22, 254.21, 239.20	tentatively identified
**2 II**	9.14	C_7_H_8_O_2_	C_29_H_50_N_14_	595.44116	595.44156	−0.68	357.25, 342.24, 316.22, 254.21, 239.20	tentatively identified
**2 III**	9.31	C_7_H_8_O_2_	C_29_H_50_N_14_	595.44104	595.44156	−0.88	357.25, 342.24, 316.22, 254.21, 239.20	tentatively identified
**3 I**	9.49	C_9_H_10_O_2_	C_31_H_52_N_14_	621.45673	621.45721	−0.79	383.27, 368.26, 316.22, 254.21, 239.20	tentatively identified
**3 II**	9.76	C_9_H_10_O_2_	C_31_H_52_N_14_	621.45734	621.45721	−0.20	383.27, 368.26, 316.22, 254.21, 239.20	tentatively identified
**4 I**	9.56	C_10_H_12_O_2_	C_32_H_54_N_14_	635.47247	635.47286	−0.62	397.28, 382.27, 316.22, 254.21, 239.20	tentatively identified
**4*^c^***	9.68	C_10_H_12_O_2_	C_32_H_54_N_14_	635.47241	635.47286	−0.71	397.28, 382.27, 316.22, 254.21, 239.20	
**5*^c^***	9.82	C_10_H_12_O_2_	C_32_H_54_N_14_	635.47241	635.47286	−0.71	397.28, 382.27, 316.22, 254.21, 239.20	
**4 IV**	10.00	C_10_H_12_O_2_	C_32_H_54_N_14_	635.47278	635.47286	−0.14	397.28, 382.27, 316.22, 254.21, 239.20	tentatively identified
**6 I**	10.21	C_12_H_14_O_2_	C_34_H_56_N_14_	661.48798	661.48851	−0.81	423.30, 408.29, 406.27, 382.27, 344.26, 330.24, 316.22, 254.21, 239.20	tentatively identified
**6 II**	10.32	C_12_H_14_O_2_	C_34_H_56_N_14_	661.48804	661.48851	−0.72	423.30, 408.29, 406.27, 382.27, 344.26, 330.24, 316.22, 254.21, 239.20	tentatively identified
**6 III**	10.53	C_12_H_14_O_2_	C_34_H_56_N_14_	661.48792	661.48851	−0.91	423.30, 408.29, 406.27, 382.27, 344.26, 330.24, 316.22, 254.21, 239.20	tentatively identified
**7 I**	10.80	C_14_H_16_O_2_	C_36_H_58_N_14_	687.50385	687.50416	−0.46	449.32, 434.30, 432.29, 344.25, 330.24, 316.22, 239.20	tentatively identified
**7 II**	11.08	C_14_H_16_O_2_	C_36_H_58_N_14_	687.50372	687.50416	−0.64	449.31, 434.30, 432.28, 344.26, 330.24, 316.22, 254.21, 239.20	tentatively identified
**8 I**	10.93	C_15_H_18_O_2_	C_37_H_60_N_14_	701.51917	701.51981	−0.93	463.33, 448.32, 446.30, 406.27, 384.29, 344.25, 330.24, 316.22, 254.21, 239.20	tentatively identified
**8*^c^***	11.03	C_15_H_18_O_2_	C_37_H_60_N_14_	701.51923	701.51981	−0.84	463.33, 448.32, 446.30, 406.27, 384.29, 344.25, 330.24, 316.22, 254.21, 239.20	
**8III**	11.19	C_15_H_18_O_2_	C_37_H_60_N_14_	701.51990	701.51981	0.12	463.33, 448.32, 406.27, 384.29, 330.24, 316.22, 239.20	tentatively identified
**8 IV**	11.45	C_15_H_18_O_2_	C_37_H_60_N_14_	701.51984	701.51981	0.03	463.33, 448.32, 446.30, 384.29, 382.27, 344.25, 330.24, 316.22, 254.21, 239.20	tentatively identified
**9 I**	11.53	C_17_H_20_O_2_	C_39_H_62_N_14_	727.53485	727.53546	−0.84	489.34, 474.33, 472.32, 382.27, 368.26, 344.26, 330.24, 316.22, 254.21, 239.20	tentatively identified
**9 II**	11.92	C_17_H_20_O_2_	C_39_H_62_N_14_	727.53491	727.53546	−0.76	489.34, 474.33, 472.32, 382.27, 368.26, 344.26, 330.24, 316.22, 254.21, 239.20	tentatively identified
**10**	11.99	C_19_H_22_O_2_	C_41_H_64_N_14_	753.55029	753.55111	−1.09	515.36, 500.35, 422.30, 408.29, 382.27, 368.26, 356.26, 344.27, 330.24, 316.22, 254.21, 239.20	tentatively identified
**11^c^**	12.21	C_20_H_24_O_2_	C_42_H_66_N_14_	767.56537	767.56676	−1.82	529.38, 514.36, 512.35, 474.33, 450.33, 433.30, 408.29, 396.29, 384.29, 382.27, 368.26, 344.26, 330.24, 316.22, 254.21, 239.20	
**11 II**	12.37	C_20_H_24_O_2_	C_42_H_66_N_14_	767.56525	767.56676	−1.98	529.37, 514.37, 450.33, 396.28, 384.29, 344.25, 330.24, 316.22, 239.20	tentatively identified
**11 III**	12.67	C_20_H_24_O_2_	C_42_H_66_N_14_	767.56689	767.56676	0.17	529.38,514.36, 316.22, 239.20	tentatively identified
**12 I**	12.64	C_22_H_26_O_2_	C_44_H_68_N_14_	793.58209	793.58241	−0.41	555.39, 540.38, 538.36, 476.35, 462.33, 422.30, 410.30, 382.27, 368.25, 344.26, 330.24, 316.22, 254.21, 239.20	tentatively identified
**12 II**	12.99	C_22_H_26_O_2_	C_44_H_68_N_14_	793.58215	793.58241	−0.33	555.38, 540.38, 316.22, 239.20	tentatively identified
**13**	13.04	C_24_H_28_O_2_	C_46_H_70_N_14_	819.59717	819.59806	−1.09	581.41, 566.40, 564.38, 474.33, 422.30, 344.26, 330.24, 316.23, 254.21, 239.20	tentatively identified

a I, II, III, and IV stand for DIAL isomers.

b Tentatively identified compounds are identified on the basis of LC−MS and LC−MS/MS data.

c Compounds have been identified on the basis of reference compounds.

### Method Validation for the Quantitation of DIALs from Tomato Fruits

**Figure 5:**
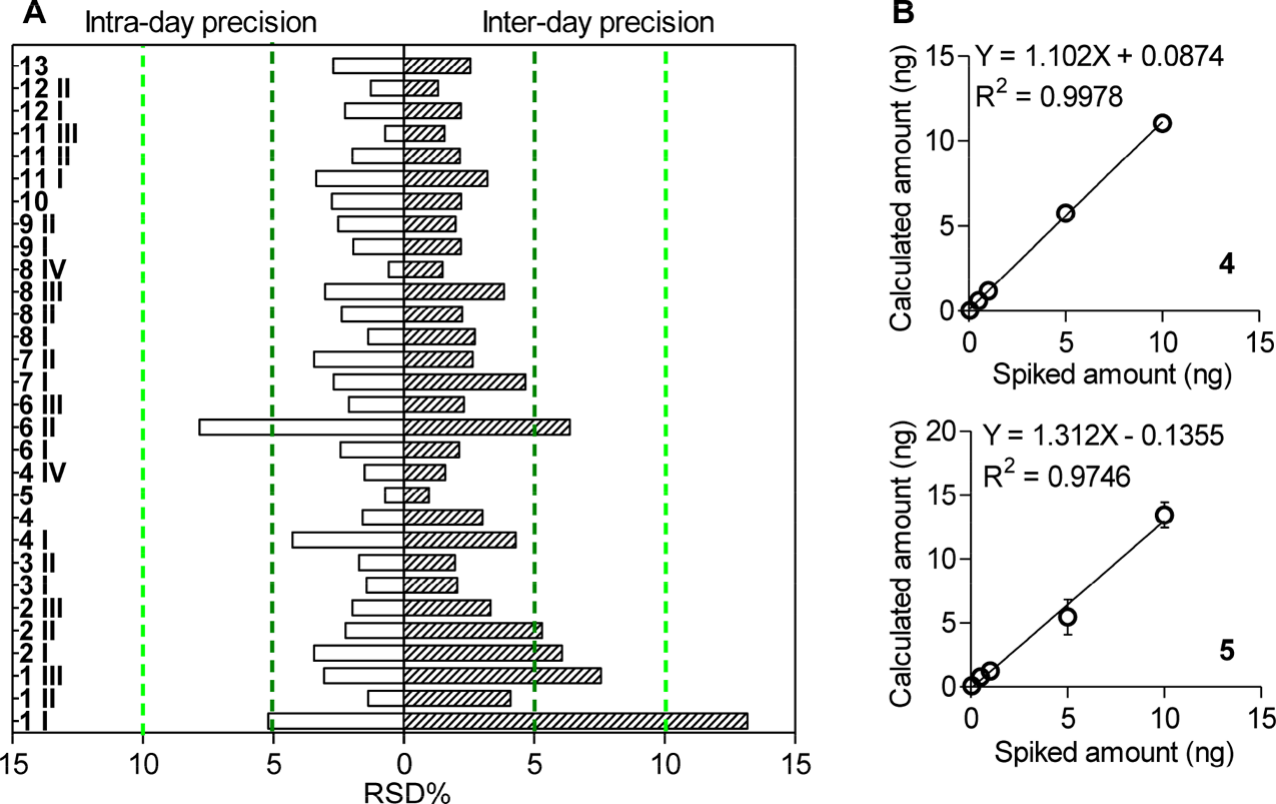
Quantitative method validation with tomato samples: (A) intra- (*n* = 5) and interday (*n* = 9) precision; (B) correlation between the added and determined amount of 4 and 5 in tomato samples. *n* = 3 in each level. Mean ± SD.

As shown in Figure S5, a coefficient of determination (*r*^2^) higher than 0.997 was obtained, indicating high linearity within the wide concentration range (covering 4 orders of magnitude) expected for each DIAL in tomato samples. LOD and LOQ for DIALs detected by MS as 0.05 and 0.1 pg/mg dry weight (DW) in tomato samples, respectively. The mean recovery percentage for *D*_6_-4 was 89.44%. As a consequence, the proposed method possesses the significant sensitivity and acceptable recovery to detect DIALs in plants and food vegetables. Intraday precision (repeatability) results for DIALs of a real lyophilized ground tomato sample showed RSD values lower than 5% for all DIALs quantified except for 1 I (5.2%) and 6 II (7.8%). Interday precision (reproducibility) results showed that RSD values for the quantitation of DIALs were lower than 5% for more than 83% of endogenous DIALs (Figure 5A). As shown in Figure 5B, the correlation between the added and determined amounts of 4 (*r*^2^ = 0.9978) and 5 (*r*^2^ = 0.9746), demonstrates a good applicability and accuracy of our approach for quantitation of DIALs.^29^ All results indicated that our analytical approach is acceptable for the quantitation of endogenous DIALs.

### Quantitative Profiling of DIALs in Vegetables

**Figure 6:**
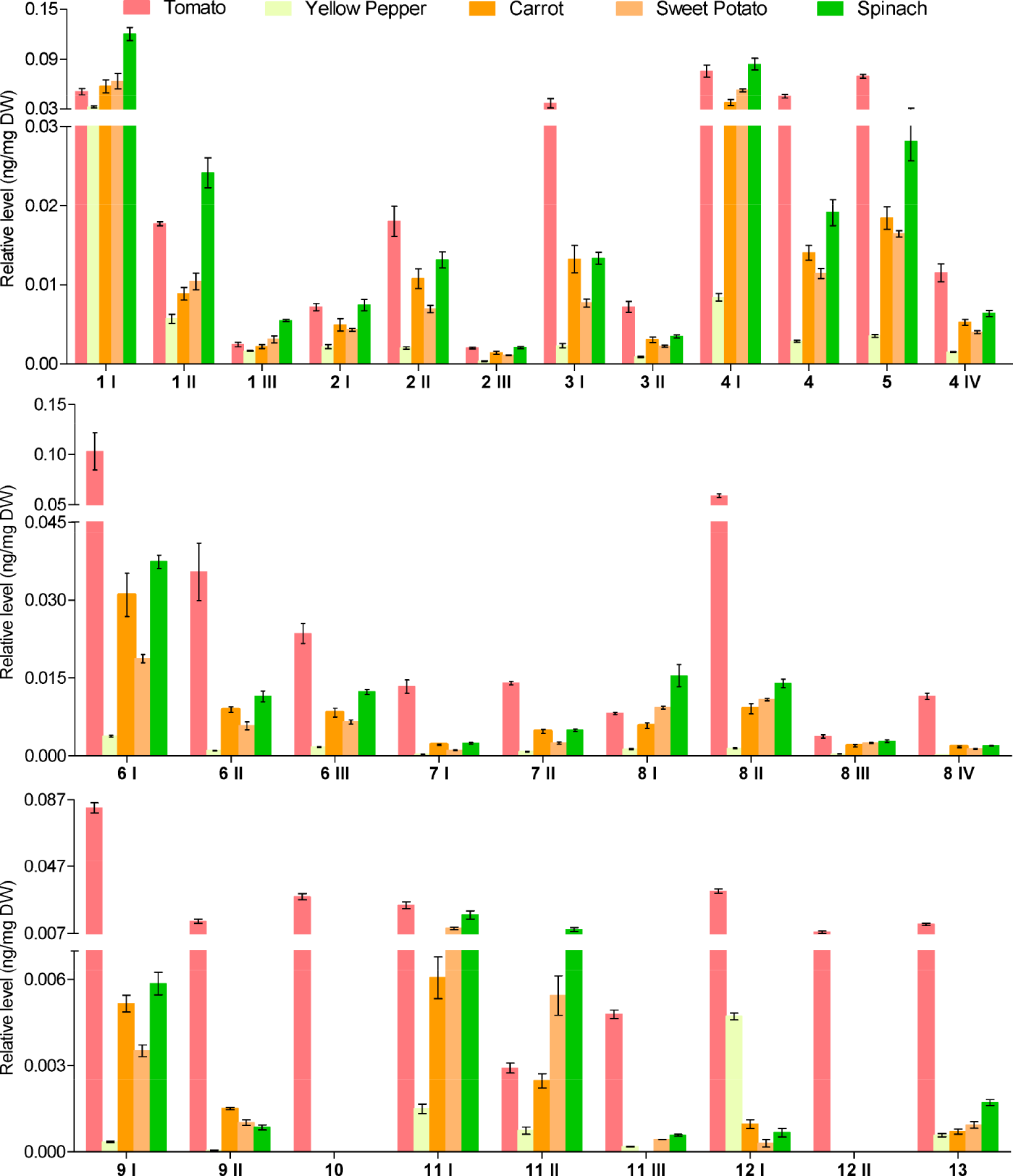
Quantitation of DIALs from vegetables. *n* = 4 in each group (*n* = 3 for tomato). Mean ± SD.

Quantitative results (Figure 6) showed that the DIALs content varied from 0.18 pg/mg (11 III in yellow pepper) to 0.12 ng/ mg DW (1 I in spinach). Among which, the levels of 4 DIALs including 8 IV, 10, 11 III, and 12 II were in carrot, yellow pepper, sweet potato, and spinach lower than LOQ, while the relative contents of more than 50% of DIALs with short carbon chains (C_5_−C_10_) and 6 I (middle length of carbon chain, C_12_) were higher than 0.01 ng/mg DW in almost all analyzed food samples. Comparison of DIALs’ levels among different food vegetables showed that tomato has the highest content in DIALs (except for 1s, 8 I, and 11 II), followed by spinach, carrot and sweet potato, and finally yellow pepper. These significant differences in DIALs amounts may correspond to the amounts of their precursor and be strongly related to the growth conditions of the analyzed vegetable species. For example, tomato has the highest content of lycopene and is exposed to more light, compared to sweet potato and carrot, indicating that lycopene may make important contributions to the formation of DIALs. In addition, vegetables and fruits may also differ in their capability to cope with oxidation processes that trigger carotenoid degradation.

In the present study, a highly sensitive and accurate chemical derivatization−UHPLC−MS strategy was developed based on silica gel SPE purification, for comprehensive characterization of DIALs. With the efficient SPE purification, mild derivatization protocol and optimized UHPLC-MS approach, DIALs at a concentration as low as 0.05 pg/mg dry vegetables material were detected. Furthermore, our method was successfully used to investigate DIALs, which may have an impact on human health, and profiled 30 endogenous members of this class of compounds with all theoretical lengths of carbon chains (C_5_−C_24_) in frequently consumed vegetables. Our innovative method will be a valuable tool for in-depth study of the impact of food DIALs on human health and paves the way for investigating the biological functions and metabolism of these compounds in plants.

## ASSOCIATED CONTENT

### Supporting Information

T3-derivatization of carotenoid-derived DIALs; optimization of T3-derivatized DIALs MS detection; T3-derivatization of apocarotenoids; confirmation of the assignment of endogenous carotenoid-derived DIALs with standards and UHPLC chromatographic retention time rule; linearity of the quantitative approach (Figures S1−S5) (PDF)

## ABBREVIATIONS USED

DIAL, dialdehyde; T3, N2,N2,N4,N4-tetraethyl-6-hydrazinyl-1,3,5-triazine-2,4-diamine; UHPLC-MS, ultrahigh performance cleavage oxygenases; GC−MS, gas chromatography-mass spectrometry; MeOH, methanol; ACN, acetonitrile; IPA, 2-propanol; FA, formic acid; BHT, butylated hydroxytoluene; F1, fraction 1; F2, fraction 2; LOD, limit of detection; LOQ, limit of quantitation; RSDs, relative standard deviations; DW, dry weight; EICs, extracted ion chromatograms; TICs, total ion chromatograms liquid chromatography-mass spectrometry; CCDs, carotenoid cleavage dioxygenases; ACO, apocarotenoid
