# Supplementary material for: A Highly Sensitive SPE Derivatization–UHPLC–MS Approach for Quantitative Profiling of Carotenoid-Derived Dialdehydes from Vegetables
Source: AIDS Behav. 2019 May 5;67(20):5899–907. doi: 10.1021/acs.jafc.9b01749 (PMC7722347; doi:10.1021/acs.jafc.9b01749)

A Highly Sensitive SPE Derivatization–UHPLC–MS Approach for Quantitative Profiling of Carotenoid-Derived Dialdehydes from Vegetables

Jianing Mi, Kun-Peng Jia, Aparna Balakrishna, Qitong Feng, and Salim Al-Babili*

King Abdullah University of Science and Technology (KAUST), Biological and Environmental Sciences and Engineering Division, The BioActives Lab, Thuwal 23955-6900, Kingdom of Saudi Arabia.

* Corresponding author

E-mail address: salim.babili@kaust.edu.sa

Figure S1. T3-derivatization of carotenoid-derived DIALs. A). Scheme of derivatization of carotenoid-derived DIALs with T3 Reagents; B). Optimization of T3 derivatization conditions of DIALs; C). Comparison of the ionization efficiency of DIALs between with and without derivatization.

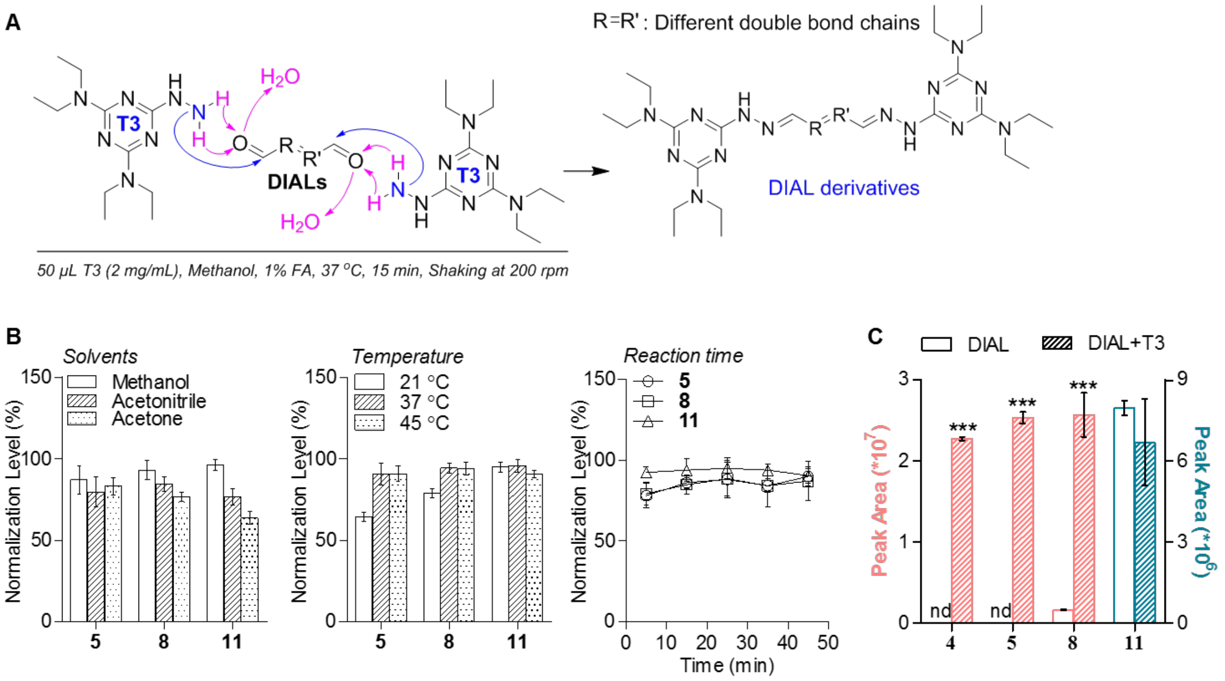

Figure S2. Optimization of T3-derivatized DIALs MS detection.

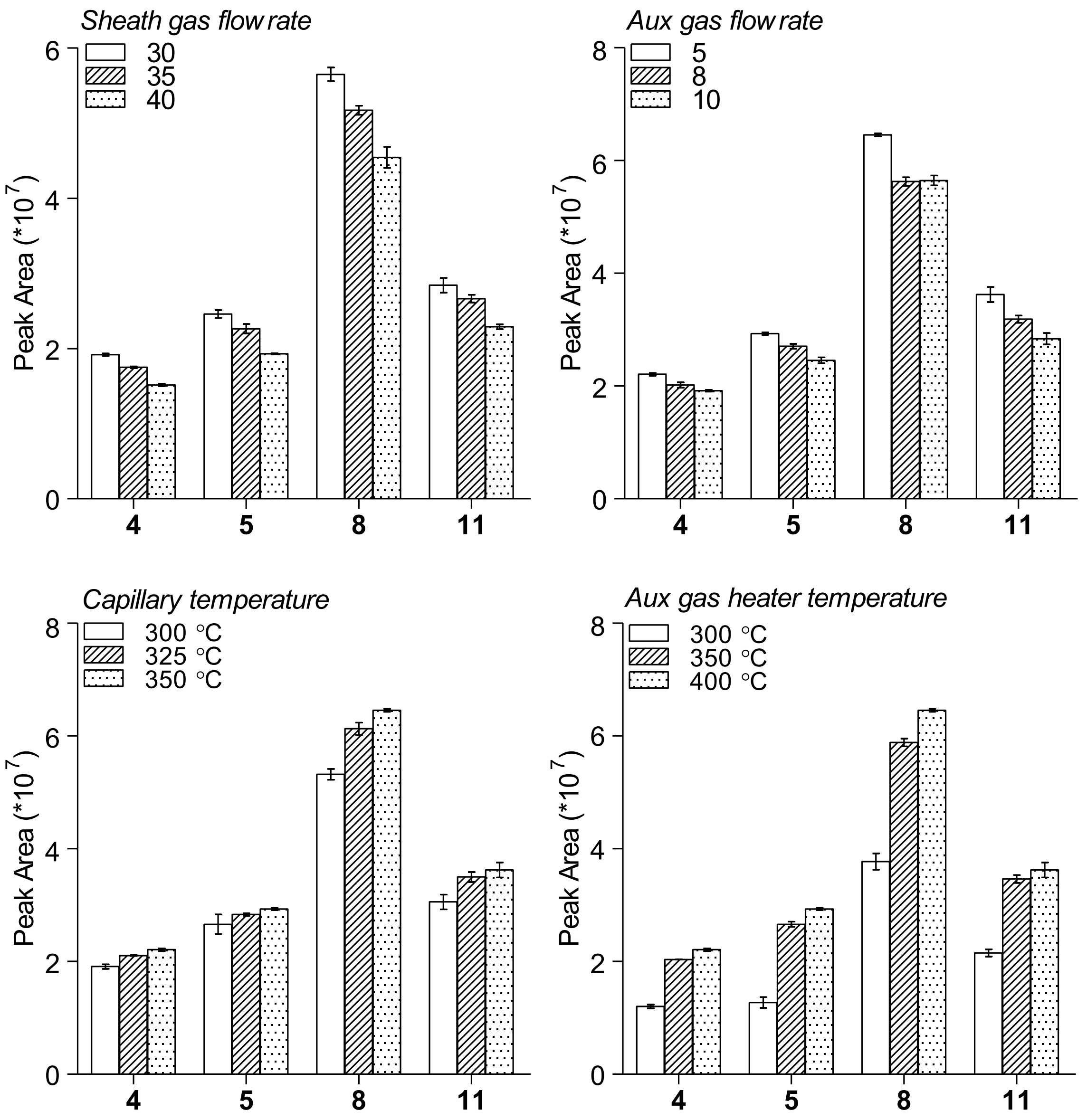

Figure S3. T3-derivatization of apocarotenoids.

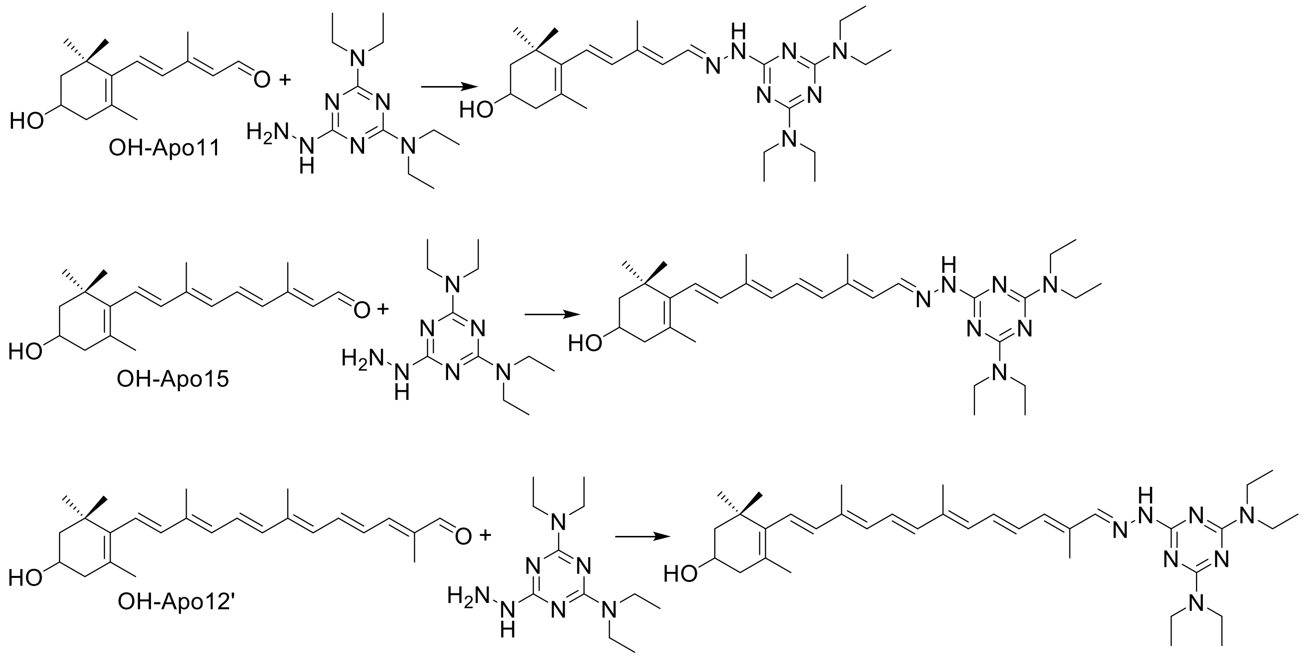

Figure S4. Confirmation of the assignment of endogenous carotenoid-derived DIALs with standards (A) and UHPLC chromatographic retention time rule (B).

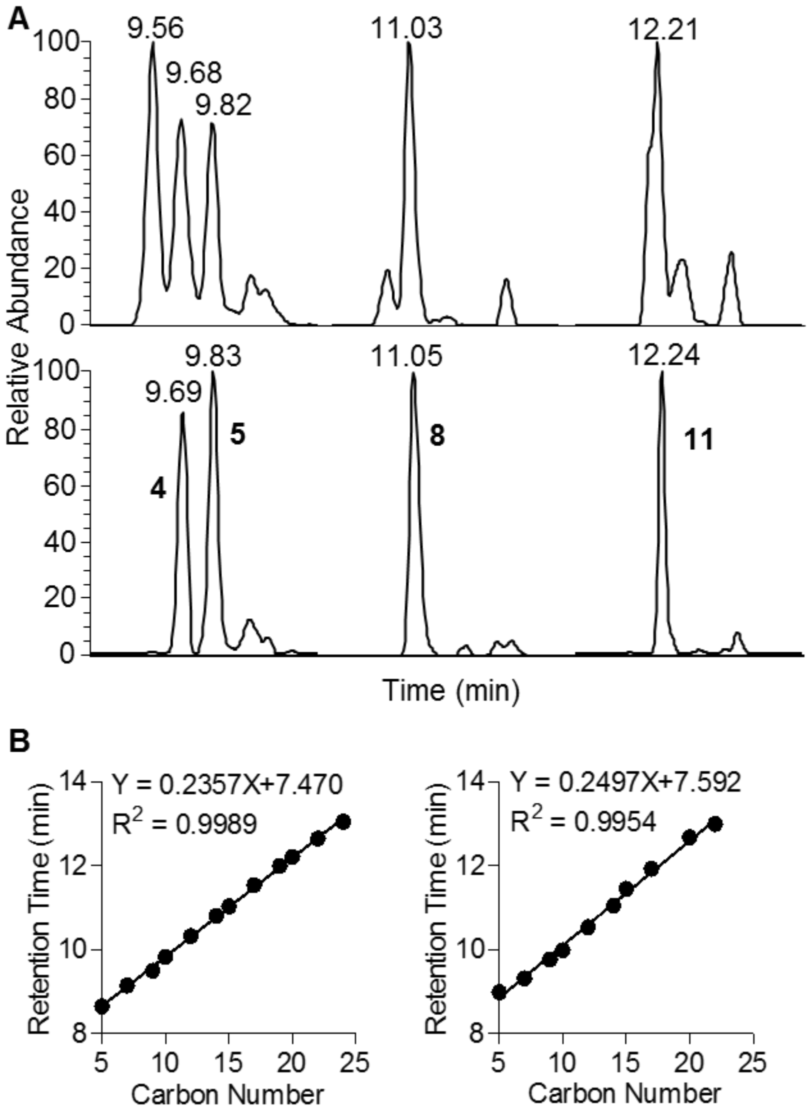

Figure S5. Linearity of the quantitative approach.

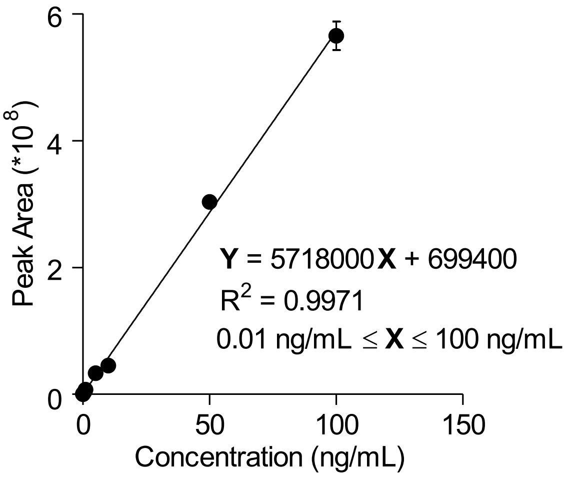

Supplement: Supplementary file 1 [file JAFC-67-20-5899-s001.pdf]
